# Clinical‐Pharmacological Drug Information Center of Hannover Medical School: Update From a Tertiary Care University Hospital (2022–2024)

**DOI:** 10.1002/prp2.70247

**Published:** 2026-04-08

**Authors:** Johannes Heck, Dirk O. Stichtenoth, Christoph Schröder, Ruxandra Sabau, Anna‐Leena K. Heim, Felix Koop, Thorben Pape, Sebastian Schröder, Martin Schulze Westhoff, Benjamin Krichevsky, Martin Klietz, Stephan Greten, Carsten Schumacher

**Affiliations:** ^1^ Institute for Clinical Pharmacology Hannover Medical School Hannover Germany; ^2^ Institute for General Practice and Palliative Care Hannover Medical School Hannover Germany; ^3^ Department of Respiratory Medicine and Infectious Diseases Hannover Medical School Hannover Germany; ^4^ Department of Psychiatry, Social Psychiatry and Psychotherapy Hannover Medical School Hannover Germany; ^5^ PRACTIS Clinician Scientist Program, Dean's Office for Academic Career Development Hannover Medical School Hannover Germany; ^6^ Interdisciplinary Emergency Department University Hospital Schleswig‐Holstein Kiel Germany; ^7^ Department of Neurology Hannover Medical School Hannover Germany; ^8^ Center for Clinical Trials Hannover Medical School Hannover Germany

**Keywords:** clinical pharmacology, drug information center, pharmacotherapy safety

## Abstract

Drug information centers (DICs) are institutions dedicated to providing independent and up‐to‐date information on medications and their usage to healthcare professionals. Here, we provide an update from the clinical‐pharmacological DIC of Hannover Medical School, covering the period from April 2022 to December 2024. In total, 438 queries were evaluated. Potential differences between patient‐specific and general queries were analyzed with Pearson's chi‐squared test or Fisher's exact test. The Holm–Bonferroni method was applied to counteract the problem of multiple comparisons. A curated selection of ten clinically interesting and educational queries is presented and discussed. 85.2% of the queries were patient‐specific, and 95.7% were submitted by physicians, predominantly internists, psychiatrists, and surgeons. Indications/contraindications, adverse drug reactions, and pharmacodynamic interactions (PDIs) represented the three most frequent query categories. Compared to our previous research, we observed increases in queries about pharmacotherapy in advanced age and drug use during pregnancy or breastfeeding. As compared to general queries, patient‐specific queries were significantly more often related to indications/contraindications (26.2% vs. 49.6%; *p* < 0.001) and PDIs (24.6% vs. 44.5%, *p* = 0.003) after adjusting for multiple comparisons. The query characteristics remained relatively stable between our previous investigation and the current analysis (2022–2024), in particular with respect to type of queries, profession of inquiring healthcare professionals, medical specialties of inquirers, and query categories. Changes mainly pertained to higher proportions of queries about pharmacotherapy in advanced age and drug use during pregnancy or breastfeeding.

**Abbreviations**
ADR: adverse drug reaction
AUC: area under the curve
AWMF: Arbeitsgemeinschaft der Wissenschaftlichen Medizinischen Fachgesellschaften (Association of the Scientific Medical Societies in Germany)
Bumps: Best Use of Medicines in Pregnancy
CDC: Centers for Disease Control and Prevention
C_max_
: maximum concentration
CYP: cytochrome P450
DI: drug information
DIC: drug information center
ESMO: European Society for Medical Oncology
GFR: glomerular filtration rate
HCQ: hydroxychloroquine
IQR: interquartile range
LDL‐C: low‐density lipoprotein cholesterol
no: number
PCSK9: proprotein convertase subtilisin/kexin type 9
PDI: pharmacodynamic interaction
PFIC: progressive familial intrahepatic cholestasis
PKI: pharmacokinetic interaction
PPI: proton pump inhibitor
RKI: Robert Koch Institute
SAP: Systems, Applications, and Products in Data Processing
SmPC: summary of product characteristics
SSNRI: selective serotonin–norepinephrine reuptake inhibitor
SSRI: selective serotonin reuptake inhibitor
TACE: transarterial chemoembolization
TDM: therapeutic drug monitoring
US: United States (of America)

## 1 Introduction

Drug information centers (DICs) are institutions dedicated to providing unbiased, independent, and up‐to‐date information on medications and their usage, communicating with various groups of healthcare professionals to enhance patient care and to promote rational drug use [1, 2, 3]. In 1962, the first DIC worldwide was established at the University of Kentucky [2, 4, 5], with many other DICs following in the United States (US) and Europe from the 1960s to 1980s [1, 2, 5, 6, 7]. Today, DICs exist in most countries around the globe, including emerging nations like Uganda [8], Ethiopia [9, 10, 11], Brazil [12], Nepal [13], and India [14, 15, 16, 17, 18].

DIC staffing and operations vary significantly between countries. In the US, DICs are typically pharmacist‐led, responding to DI queries mainly by phone [2, 19, 20]. European DICs are often headed by clinical pharmacologists or similarly qualified physicians, and are operated jointly by physicians and pharmacists [2, 6, 7, 20, 21]. However, numerous pharmacist‐led DICs also exist in Europe [2]. Due to their university hospital affiliations, European DICs also often involve visiting physicians and medical students [2, 22]. European DICs generally offer written responses alongside verbal communication with inquirers [2, 22].

A plethora of reports from DICs located in emerging countries [8, 9, 10, 11, 12, 14, 15, 16, 17, 18] and Scandinavia [6, 20, 23, 24, 25, 26] were published over the last few years; however, there was a notable shortage of studies from central Europe, particularly Germany [2]. The most recent data from German clinical pharmacologist‐led DICs dated back to Tröger and Meyer (2000) [27] and Schwarz et al. (1999) [28]. In 2022, to address this information gap, we analyzed 594 queries received by the clinical‐pharmacological DIC of Hannover Medical School, a tertiary care university hospital in northern Germany, between October 2018 and April 2022 [2]. We found that the majority of queries were submitted by physicians (96.1%), predominantly internists (31.1%) [2]. 82.8% of the queries were patient‐specific, as opposed to 17.2% general queries [2]. Adverse drug reactions (ADRs), indications/contraindications, and pharmacodynamic interactions (PDIs) represented the three most frequent query categories [2]. Compared to general queries, patient‐specific queries more often addressed ADRs, PDIs, and pharmacokinetic interactions (PKIs) [2].

In 1999, Schwarz and co‐workers posited that “the periodical analysis of the types and sources of enquiries may point to latent problems and needs in the medical community” [28], a statement that is still valid today. Therefore, we here provide an update from our DIC that covers the period from April 2022 to December 2024. We sought to compare the characteristics of DI queries with our previous investigation [2] and other German as well as international studies. We aimed to examine differences between patient‐specific and general queries and sought to delineate potential changes over time. Additionally, we present and discuss a curated selection of clinically complex and educational queries our DIC dealt with during the study period.

## 2 Methods

### 2.1 Ethics Statement

This study was approved by the Ethics Committee of Hannover Medical School (No. 11267_BO_K_2024) and adheres to the Declaration of Helsinki (1964) and its later amendments (current version dating from 2024).

### 2.2 Operational Procedures of the Clinical‐Pharmacological Drug Information Center of Hannover Medical School

The operational procedures of the clinical‐pharmacological DIC of Hannover Medical School were elaborated in detail in our publication from 2022 [2]. In brief, the DIC of Hannover Medical School is a specialist pharmacotherapeutic consultation service for healthcare professionals at Hannover Medical School and affiliated academic teaching hospitals. The DIC is staffed by two senior physicians (a clinical pharmacologist and an internist), a resident in clinical pharmacology, and a clinical pharmacist. Healthcare professionals submit queries to the DIC through the hospital information system SAP (Systems, Applications, and Products in Data Processing, Walldorf, Germany) or via e‐mail. The resident or clinical pharmacist handles these queries, occasionally aided by visiting physicians or medical students. If important clinical data (e.g., about the patient's medication or comorbidities) is lacking, the inquirer is contacted for additional information. Subsequently, a thorough literature review is conducted, utilizing summaries of product characteristics (SmPCs), medical databases, current editions of standard textbooks of pharmacology and clinical pharmacology, as well as drug information and interaction software (e.g., AiDKlinik (Dosing GmbH, Heidelberg, Germany)). A written response is prepared, reviewed, countersigned by a senior physician, and delivered to the inquirer within one to three workdays. In urgent cases, a preliminary response is communicated by phone. To maintain quality standards, all queries and responses are presented and discussed at weekly pharmacotherapeutic case conferences, which are also attended by external physicians and medical students.

### 2.3 Data Acquisition

For the current update, all queries submitted to the clinical‐pharmacological DIC of Hannover Medical School between 1 April 2022 and 31 December 2024 were examined. Similar to our previous publication [2], the following parameters were analyzed for each query:

- Origin of the query: Hannover Medical School; academic teaching hospital; private practice; other.
- Type of query: patient‐specific (i.e., queries concerning individual patients) or general (i.e., queries of common pharmacological interest or queries concerning more broadly defined patient populations such as pregnant women).
- Age and sex of the patient (assessed solely for patient‐specific queries)
- Medical specialty of the inquiring healthcare professional.
- Query categories (assignment of more than one category per query was possible): adverse drug reaction (ADR); indication/contraindication; posology/dose adjustment (e.g., due to renal or hepatic insufficiency); therapeutic drug monitoring (TDM); pharmacogenetics; pharmacodynamic interaction (PDI); pharmacokinetic interaction (PKI); pregnancy and breastfeeding; pharmacotherapy in advanced age (i.e., patients ≥ 65 years); other.

Of note, the category “pharmacotherapy in advanced age” was not directly assigned to all queries concerning patients of chronological age ≥ 65 years, but solely to queries that explicitly addressed pharmacotherapeutical aspects in older individuals.

### 2.4 Statistical Analysis

Descriptive statistical methods were used to summarize the data. Quantitative variables are displayed as means ± standard deviations or as medians with interquartile ranges (IQRs) for data that were not normally distributed. Categorical variables are depicted as absolute and relative frequencies. Differences between general and patient‐specific queries were analyzed with Pearson's chi‐squared test or Fisher's exact test, as appropriate. The latter was preferred if at least one of the four cells of a 2 × 2 contingency table had an expected frequency of less than five observations. Fundamentally, *p*‐values < 0.05 were considered statistically significant. To address the problem of multiple comparisons and to reduce the likelihood of type I errors, the Holm–Bonferroni correction method was applied. All statistical analyses were performed with Microsoft Excel 2019 (Redmond, Washington, USA) and IBM SPSS Statistics for Windows, version 29 (Armonk, New York, USA).

## 3 Results

### 3.1 Number, Type, and Origin of Queries

In total, 438 queries were received by the DIC during the study period. Of these, 85.2% were patient‐specific, whereas the remaining 14.8% were general queries (Table 1). The majority of queries were submitted by physicians (95.7%). 85.2% of queries came from healthcare professionals working at Hannover Medical School. Internists, psychiatrists, and surgeons most frequently consulted the clinical‐pharmacological DIC, submitting 28.5%, 17.6%, and 16.0% of queries, respectively.

**TABLE 1:** Characteristics of queries received by the clinical‐pharmacological drug information center of Hannover Medical School between April 2022 and December 2024 (*n* = 438).

Variables	No. of queries	%
Type of query		
Patient‐specific	373	85.2
General	65	14.8
Origin of query		
Hannover Medical School	373	85.2
Academic teaching hospital	28	6.4
Private practice	15	3.4
Other (e.g., medical laboratories, agencies, medical students not affiliated with Hannover Medical School, retired physicians, etc.)	22	5.0
Profession of inquiring healthcare professional		
Physician	419	95.7
Dentist	4	0.9
Other (e.g., medical student)	15	3.4
Specialty of inquiring healthcare professional		
Internal medicine	125	28.5
Psychiatry and psychosomatic medicine	77	17.6
Surgery	70	16.0
Urology	41	9.4
Gynecology and obstetrics	40	9.1
Neurology	17	3.9
Pediatrics	13	3.0
Radiology and radiotherapy	12	2.7
Other	37	8.4
Not documented	6	1.4

Abbreviation: no., number.

### 3.2 Patient Characteristics

**FIGURE 1:**
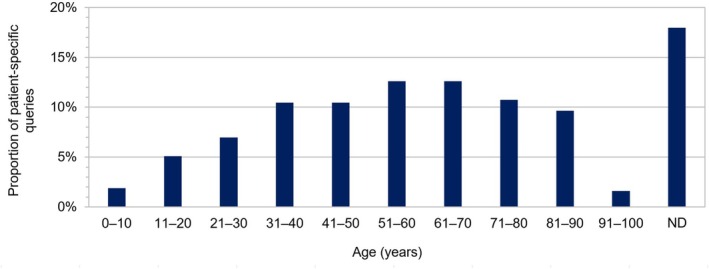
Age distribution of patient‐specific queries (*n* = 373) received by the clinical‐pharmacological drug information center of Hannover Medical School between April 2022 and December 2024. ND, not documented.

Age and sex of patients were documented in 82.0% (306/373) and 97.3% (363/373) of patient‐specific queries, respectively. The mean age of the patients was 54.0 ± 22.4 years (range 1 to 99 years). 60.3% (225/373) of the patients were female (male: 37.0% (138/373); sex not documented: 2.7% (10/373)). Patient‐specific queries most frequently concerned patients aged between 51 and 70 years (Figure 1).

### 3.3 Query Categories

Indications/contraindications, ADRs, and PDIs represented the three most frequently addressed query categories, being involved in 46.1%, 42.5%, and 41.6% of all queries, respectively (Table 2). Absolute and relative frequencies of query categories, stratified by specialties of inquiring healthcare professionals, are displayed in Table S1. In relative terms, ADRs and indications/contraindications were most frequently addressed by radiologists/radiotherapists (100% (12/12) and 66.7% (8/12) of all queries from radiologists/radiotherapists, respectively), while queries concerning posology/dose adjustments and TDM were most frequently submitted by urologists (58.5% (24/41) and 24.4% (10/41), respectively). PDIs, PKIs, and pharmacotherapy during pregnancy and breastfeeding were most commonly referred to by gynecologists (62.5% (25/40), 57.5% (23/40), and 32.5% (13/40), respectively). Pharmacogenetic topics were most frequently inquired by pediatricians (15.4% (2/13)), whereas pharmacotherapy in advanced age was referred to in approximately every third query by surgeons (31.4% (22/70)).

**TABLE 2:** Absolute and relative frequencies of query categories involved in queries received by the clinical‐pharmacological drug information center of Hannover Medical School between April 2022 and December 2024.

Query category	Total (*n* = 438)	Patient‐specific queries (*n* = 373; 85.2%)	General queries (*n* = 65; 14.8%)	*p*‐value (in ascending order)	*i*	Threshold *α*_i_ for statistical significance according to Holm–Bonferroni method (*α*_i_ = *α*/(m—*i* + 1) with *α* = 0.05 and m = 10)	*p*‐value statistically significant after adjustment for multiple comparisons?
Indication/contraindication—no. (%)	202 (46.1)	185 (49.6)	17 (26.2)	< 0.001^a^	1	0.005	Yes
Pharmacodynamic interaction—no. (%)	182 (41.6)	166 (44.5)	16 (24.6)	0.003^a^	2	0.006	Yes
Posology/dose adjustment (e.g., due to renal or hepatic insufficiency)—no. (%)	101 (23.1)	94 (25.2)	7 (10.8)	0.011^a^	3	0.006	No
Other—no. (%)	53 (12.1)	39 (10.5)	14 (21.5)	0.011^a^	4	0.007	No
Adverse drug reaction—no. (%)	186 (42.5)	167 (44.8)	19 (29.2)	0.019^a^	5	0.008	No
Therapeutic drug monitoring—no. (%)	52 (11.9)	49 (13.1)	3 (4.6)	0.050^a^	6	0.010	No
Pharmacokinetic interaction—no. (%)	176 (40.2)	157 (42.1)	19 (29.2)	0.051^a^	7	0.013	No
Pharmacotherapy in advanced age—no. (%)	40 (9.1)	38 (10.2)	2 (3.1)	0.066^a^	8	0.017	No
Pharmacogenetics—no. (%)	21 (4.8)	20 (5.4)	1 (1.5)	0.340^b^	9	0.025	No
Pregnancy and breastfeeding—no. (%)	27 (6.2)	25 (6.7)	2 (3.1)	0.402^b^	10	0.050	No

*Note:* Assignment of more than one category per query was possible. The Holm–Bonferroni method was applied to address the problem of multiple comparisons and to reduce the likelihood of type I errors. To this end, the table was sorted in order of ascending *p*‐values (as denoted by the counter variable *i*), and step‐specific thresholds *α*_i_ to determine statistical significance were calculated. The overall significance level *α* was set at 0.05, and the number of comparisons was m = 10.

Abbreviation: no., number.

^a^ Pearson's chi‐squared test.

^b^ Fisher's exact test.

### 3.4 Comparison Between General and Patient‐Specific Queries

As compared to general queries, patient‐specific queries were more often related to ADRs (29.2% vs. 44.8%; *p* = 0.019), indications/contraindications (26.2% vs. 49.6%; *p* < 0.001), posology/dose adjustment (10.8% vs. 25.2%; *p* = 0.011), TDM (4.6% vs. 13.1%; *p* = 0.050), and PDIs (24.6% vs. 44.5%; *p* = 0.003) before adjusting for multiple comparisons (Table 2). By contrast, the query category “other” was more often addressed by general queries as compared to patient‐specific queries (21.5% vs. 10.5%; *p* = 0.011). After adjustment for multiple comparisons using the Holm–Bonferroni correction method, only the differences between general and patient‐specific queries with respect to indications/contraindications and PDIs remained statistically significant.

### 3.5 Examples of Clinically Complex Queries

To illustrate the clinical complexity of DI queries answered by our DIC, we present and comment on a curated selection of ten queries (Table 3). Those queries (and corresponding answers) were selected subjectively by the authors based on their potential clinical interest and educational value to readers. The selected cases are not intended to be representative of the entire sample of queries analyzed in this study; they only serve illustrative and educational purposes. Three of the ten selected queries were (primarily) related to the pharmacological treatment of rare diseases (cases no. 2, 7, and 10), two queries dealt with drug indications/contraindications (cases no. 5 and 8), two queries pertained to the management of drug interactions (cases no. 1 and 6), and one query each referred to pharmacotherapy during pregnancy (case no. 3), pharmacotherapy in advanced age (case no. 4), and pharmacogenetics (case no. 9).

**TABLE 3:** Examples of queries answered by the clinical‐pharmacological drug information center of Hannover Medical School between April 2022 and December 2024.

Case no.	Question	Answer	Comment
1	“A 42‐year‐old male patient is being treated with two tyrosine kinase inhibitors—ponatinib and asciminib—for chronic myeloid leukemia with blast crisis. He has now developed gastritis and necessitates therapy with a proton pump inhibitor (PPI), preferably pantoprazole. Does pantoprazole interact with ponatinib and/or asciminib?”	“According to the Summary of Product Characteristics (SmPC), co‐administration of ponatinib with a potent inhibitor of gastric acid secretion leads to a minor (approximately 25%) reduction in the maximum concentration (C_max_) of ponatinib, with no impact on the area under the curve (AUC). The European Society for Medical Oncology (ESMO) concludes that PPIs may be administered concurrently with ponatinib For asciminib, the SmPC does not provide such information. However, a study by Hoch et al. demonstrated similar outcomes when combining asciminib and rabeprazole, resulting in only a slight (approximately 9%) reduction in C_max_ and no significant decrease in AUC. The co‐administration of ponatinib and/or asciminib with a PPI does not lead to a clinically relevant reduction in absorption”	The resorption of certain tyrosine kinase inhibitors (e.g., erlotinib and dasatinib) is reduced by concomitant administration of PPIs. This case highlights the necessity to assess each drug within the same medication class individually, since there may exist significant pharmacokinetic differences
2	“One of our patients is suffering from progressive familial intrahepatic cholestasis (PFIC) type 1, causing significantly elevated cholestasis parameters (liver transaminases around 500 U/L). His current medication comprises ursodeoxycholic acid, odevixibat, dronabinol drops, and, possibly, rifampicin in the near future. The patient is currently experiencing considerable mental distress and is suffering from severe sleep disorder. Is there a medication with a sleep‐inducing effect that does not further elevate the patient's liver transaminases? I am considering prescribing a low‐potency antipsychotic (e.g., pipamperone) or, alternatively, a benzodiazepine”	“We would opt for pipamperone as the most suitable candidate drug in this specific case. Elevations of liver transaminases or cholestasis parameters are generally not a clinically significant concern with pipamperone. In addition, pipamperone does not interfere with the planned use of rifampicin. Melperone, on the other hand, has been associated with increases in liver enzymes, intrahepatic cholestasis, and jaundice, making it a less well‐suited alternative. If a benzodiazepine is preferred, temazepam is the most suitable choice because, unlike lorazepam, it does not interact with rifampicin”	This case demonstrates the importance of considering potential drug–drug interactions not only within the current medication but also with drugs that are likely to be administered in the near future. For instance, common sleep‐inducing drugs such as zopiclone, zolpidem, daridorexant, and melatonin may interact with rifampicin, potentially leading to a reduction or loss of their effect
3	“A 30‐year‐old pregnant patient is suffering from sickle cell anemia. Currently, her hemoglobin level is < 7 g/dL and her ferritin level is 500 ng/dL. The patient refuses blood transfusions for religious reasons. I would like to ask for your evaluation regarding potential treatment options with voxelotor and/or erythropoietin during pregnancy”	“We consider the administration of erythropoietin (which does not cross the placenta) during pregnancy to be acceptable if the patient's blood pressure is monitored closely. Studies have shown no adverse effects of erythropoietin therapy during pregnancy. On the other hand, there is very limited experience with the use of voxelotor during pregnancy. Animal studies did not show teratogenic effects. However, due to a lack of data from humans, the SmPC of voxelotor advises against its use in pregnancy”	Pharmacotherapy during pregnancy poses a significant challenge as many drugs lack a comprehensive evidence base. Careful selection of medications with established safety profiles, transparent communication with patients, and vigilant monitoring are essential aspects of ensuring optimal outcomes for both mother and child
4	“A 79‐year‐old female patient who underwent transarterial chemoembolization (TACE) is now experiencing nausea and vomiting. Additionally, the patient has angle‐closure glaucoma. The patient's current medication comprises bisoprolol, candesartan, apixaban, simvastatin, torasemide, metamizole, lactulose, sodium picosulfate, as well as brimonidine, dorzolamide, and latanoprost–timolol for topical application to the eye. Unfortunately, her current antiemetic therapy with metoclopramide is not sufficient. Are there any recommendations for further pharmacological antiemetic treatment considering the patient's angle‐closure glaucoma? Can we switch from metoclopramide to alizapride, for example?”	“Yes, in principle it is possible to switch from metoclopramide to alizapride or domperidone in this specific case if the current therapy with metoclopramide is insufficient. However, metoclopramide, domperidone as well as alizapride exert similar pharmacodynamic effects, i.e., antagonism of dopamine receptors. Therefore, it is doubtful whether a significant improvement can be achieved by switching within the same drug class. Additionally, anti‐dopaminergic medications should be used with caution in older individuals due to the risk of extrapyramidal motor symptoms. Co‐administration of two anti‐dopaminergic antiemetics (e.g., metoclopramide plus alizapride) should be avoided. Possible alternatives to alizapride might be a 5‐HT_3_ receptor antagonist (e.g., ondansetron) or an NK_1_ receptor antagonist (e.g., (fos)aprepitant). Both drug classes can be combined with metoclopramide or also with each other if necessary. It should be noted that (fos)aprepitant is a moderate inhibitor of CYP3A4 and may increase the plasma levels of apixaban, bisoprolol, and simvastatin—we recommend increased vigilance for adverse drug reactions. Dexamethasone should be avoided due to the risk of deterioration of the patient's angle‐closure glaucoma. Dimenhydrinate and scopolamine are contraindicated in patients with glaucoma”	Antiemetic pharmacotherapy in older patients with comorbidities is challenging due to altered organ functions, increased susceptibility to adverse drug reactions (e.g., increased risk of extrapyramidal motor symptoms caused by anti‐dopaminergic agents), and potential drug–drug interactions due to polypharmacy. Taken together, a cautious and personalized approach is required
5	“I am going to treat an outpatient in the dental clinic who is currently taking trimipramine, oxcarbazepine, lithium, and levothyroxine. I would like to use articaine as local anesthetic during a planned dental procedure. Articaine is available in different preparations, either with (e.g., Ultracain D‐S forte, Ultracain D‐S) or without adrenaline (e.g., Ultracain D). Alternatively, lidocaine or mepivacaine is available at our clinic. To my knowledge, trimipramine can interact with local anesthetics, potentially leading to adverse drug reactions, especially hypertensive crises. Would those potential hypertensive crises be due to the added adrenaline? Could I use articaine without adrenaline instead?”	“Tricyclic antidepressants delay the inactivation of catecholamines by blocking their active reuptake into presynaptic storage vesicles. Based on a literature review and the SmPC of Ultracain D‐S forte (which contains articaine plus adrenaline), the combination of tricyclic antidepressants with adrenaline is contraindicated as it may lead to hypertensive crises and ventricular arrhythmias. By contrast, the SmPC of trimipramine is less restrictive: While limiting the dose of adrenaline upon co‐administration with trimipramine is recommended (less than 0.2 mg adrenaline in 10 min or 0.3 mg in 1 h in adults), the co‐administration is not contraindicated per se. In the case of your patient, we recommend using available local anesthetics (e.g., articaine, lidocaine, or mepivacaine) without additional adrenaline”	“While drug combination products are often convenient to use, the complexity of their composition increases the likelihood of interactions between individual components and other drugs. Unfortunately, the individual drugs contained in branded combination products are often not immediately evident. In the case of articaine, the brand names Ultracain D‐S and Ultracain D differ by just one letter, and it is not directly apparent that the former contains additional adrenaline while the latter does not. In the worst case, this circumstance may be overlooked by the attending physician, leading to unexpected interactions. This underscores the importance of using international nonproprietary names during prescribing in lieu of brand names as a simple measure to avoid undesired drug–drug interactions”
6	“We have encountered discrepancies in the SmPCs of calcium channel blockers (amlodipine, lercanidipine) regarding possible co‐administration with cyclosporine. We would like to ask for your advice whether co‐administration of cyclosporine and calcium channel blockers is possible or contraindicated”	“The SmPC of lercanidipine states that concurrent use with cyclosporine is contraindicated. The pharmacokinetics of lercanidipine and cyclosporine mutually influence each other, resulting in an increase in plasma levels of both substances. The SmPC of cyclosporine also advises against co‐administration with lercanidipine (however without official contraindication) or, if co‐administration is unavoidable, it recommends administering cyclosporine at least 3 h after lercanidipine. We strongly advise against administering lercanidipine and cyclosporine together. If cyclosporine and a dihydropyridine‐type calcium channel blocker need to be administered concurrently, we recommend nitrendipine, amlodipine, felodipine, or isradipine instead of lercanidipine, with appropriate clinical (blood pressure) and plasma level controls (cyclosporine). If co‐administration of lercanidipine and cyclosporine is unavoidable, cyclosporine should be administered at least 3 h after lercanidipine to minimize mutual pharmacokinetic interactions. Given the existing contraindication in the SmPC of lercanidipine, detailed and well‐documented information of the patient is paramount in such cases. Alternatively, a switch to another antihypertensive drug class (such as angiotensin‐converting enzyme inhibitors, angiotensin receptor antagonists, or thiazide diuretics) should be considered”	Discrepancies about the same interactions in SmPCs of different drugs are not uncommon. Such discrepancies can be due to differences in SmPC update cycles, interpretations of clinical studies, or regulatory guidance, underscoring the necessity of a nuanced and individualized approach to ensure patient safety and optimal therapeutic outcomes
7	“A 29‐year‐old female patient is suffering from migraine, anxiety, and depression, along with factor VII deficiency. Which medication would be the best choice for treating her anxiety and depression, considering the patient's bleeding disorder? From a clinical‐pharmacological standpoint, is there any argument against using metamizole and/or a triptan for the patient's migraine?”	“Agomelatine, mirtazapine, bupropion, as well as tricyclic antidepressants are not associated with a clinically relevant risk of bleeding. From a clinical‐pharmacological perspective, these antidepressants are suitable options. By contrast, selective serotonin reuptake inhibitors (SSRIs) and selective serotonin–norepinephrine reuptake inhibitors (SSNRIs) should be avoided as they increase the risk of bleeding. Non‐steroidal anti‐inflammatory drugs should also be avoided in this specific case as they are associated with an increased risk of bleeding, too. According to the German AWMF (Arbeitsgemeinschaft der Wissenschaftlichen Medizinischen Fachgesellschaften) S1 guideline on the treatment of migraine, metamizole can be used to treat an acute migraine attack. Metamizole can be administered orally or intravenously, with or without additional metoclopramide. A therapy with triptans is also feasible for your patient. However, if triptans, metoclopramide, and a serotonergic antidepressant like mirtazapine or a tricyclic antidepressant are administered concurrently, the increased risk of serotonin syndrome should be taken into account”	Considering individual patient factors is crucial to ensure both safety and efficacy of pharmacological treatment. Factors such as age, sex, organ function, and comorbidities significantly influence drug choice. This case illustrates that drugs usually considered first choice (such as SSRIs in the treatment of depression) are not appropriate in every patient context, particularly if the patient is afflicted with an additional bleeding disorder
8	“A 69‐year‐old male liver transplant patient with severely impaired renal function (glomerular filtration rate (GFR) currently 24 mL/min) also displays severe atherosclerosis with 70% carotid stenosis. The patient is currently taking the following medications: cyclosporine, mycophenolate mofetil, candesartan, pantoprazole, sodium bicarbonate, and cholecalciferol. The patient's low‐density lipoprotein cholesterol (LDL‐C) target level is < 55 mg/dL. As the GFR is less than 30 mL/min, we have discontinued rosuvastatin. Is there an alternative statin suitable for co‐administration with cyclosporine? What would be the maximum dose? Adequate LDL‐C reduction is not expected with ezetimibe alone. Are there any recommendations regarding proprotein convertase subtilisin/kexin type 9 (PCSK9) inhibitor therapy in immunosuppressed patients?”	“In the case of this patient, statins should be avoided due to renal impairment and potential drug–drug interactions with cyclosporine. PCSK9 inhibitors can be considered as a suitable alternative, with a preference for evolocumab due to slightly better data regarding use in patients with renal impairment. The use of PCSK9 inhibitors in transplant patients is generally considered safe and effective, although data are limited. An alternative to PCSK9 inhibitors may be bempedoic acid”	Patients taking immunosuppressive drugs are at increased risk of drug–drug interactions. Monoclonal antibodies such as evolocumab have the advantage that, due to their pharmacokinetic properties (e.g., CYP‐independent metabolism) they are usually not prone to drug–drug interactions
9	“A 48‐year‐old patient is suffering from multiple psychiatric disorders and has reported poor tolerance to antidepressants in the past with numerous adverse drug reactions. Genetic testing has revealed that the patient is an intermediate metabolizer with respect to the cytochrome P450 (CYP) isoenzyme 2C19. The patient is currently experiencing sleep disorders under treatment with bupropion. Is it possible to switch to an SSRI considering the patient's CYP2C19 metabolizer status? Is a dose adjustment necessary?”	“SSRIs are metabolized by the following CYP isoenzymes: Sertraline: 2D6, 2C19 Citalopram/escitalopram: 2D6, 2C19 Fluoxetine: 2D6, 2C19 Fluvoxamine: 2D6 Paroxetine: 2D6 This suggests that fluvoxamine or paroxetine might be primarily considered for your patient, as they are not metabolized via CYP2C19. Of note, according to the guidelines of the Clinical Pharmacogenetics Implementation Consortium, there is no need to adjust the initial dose of any of the abovementioned SSRIs in patients with CYP2C19 intermediate metabolizer status. Regardless of the selected SSRI, we recommend monitoring clinical tolerability, especially at the start of treatment”	This case illustrates that results from pharmacogenetic testing are sometimes difficult to interpret. While fluvoxamine and paroxetine appear as the most suitable SSRIs from a pharmacokinetic view, they must also be evaluated critically to determine if they are clinically appropriate for the treatment of the patient's psychiatric disorders
10	“We have recently diagnosed a patient with chronic Q fever presenting with endocarditis. The Centers for Disease Control and Prevention (CDC) recommends a treatment regimen of 100 mg doxycycline every 12 h together with 200 mg hydroxychloroquine (HCQ) every 8 h for up to 24 months. The CDC recommendation for the HCQ dose is apparently not weight‐adjusted, and it is notably higher than the maximum HCQ dosage of 5 mg/kg body weight proposed by rheumatological guidelines. The weight of our patient is 60 kg; is it hence appropriate to administer HCQ in such a high dosage for an extended period of time?”	“For the treatment of endocarditis due to chronic infection with *Coxiella burnetii* (chronic Q fever), the CDC recommends a combination of doxycycline (2 × 100 mg per day) and HCQ (3 × 200 mg per day) for 18 months (native heart valves) or 24 months (prosthetic valves). The German Robert Koch Institute (RKI) suggests a combination therapy of doxycycline with either a fluoroquinolone (group 3 or 4) or rifampicin, or—alternatively—a combination of doxycycline and (hydroxy)chloroquine for at least 12 months. For your patient with a body weight of 60 kg, the HCQ dosage for the treatment of chronic Q fever corresponds to 10 mg/kg. While this exceeds the maximum recommended body weight‐adjusted dose of HCQ proposed by rheumatological guidelines for the long‐term treatment of rheumatic diseases (i.e., ≤ 5 mg/kg), the treatment duration of chronic Q fever is considerably shorter: 18–24 months compared to potentially lifelong treatment. It is advised to perform therapeutic drug monitoring to assess plasma levels of HCQ (target plasma level 0.8–1.2 μg/mL) and doxycycline (target plasma level ≥ 5 μg/mL) during treatment of chronic Q fever. Additionally, the eye fundus should be examined regularly. Since both doxycycline and HCQ increase the photosensitivity of the skin, adequate sun protection is also critical”	HCQ is used in rheumatology for its immunomodulatory properties, such as interaction with toll‐like receptors. In the case of Q fever, HCQ is employed to elevate the pH level of phagosomes, in which *Coxiella burnetii* persists. In combination with doxycycline, a bactericidal effect is achieved. A higher body weight‐based dosage of HCQ in the treatment of chronic Q fever compared to rheumatic diseases is possible because the treatment duration is much shorter

Abbreviations: AUC, area under the curve; AWMF, Arbeitsgemeinschaft der Wissenschaftlichen Medizinischen Fachgesellschaften (Association of the Scientific Medical Societies in Germany); CDC, Centers for Disease Control and Prevention; C_max_, maximum concentration; CYP, cytochrome P450; ESMO, European Society for Medical Oncology; GFR, glomerular filtration rate; HCQ, hydroxychloroquine; LDL‐C, low‐density lipoprotein cholesterol; no., number; PCSK9, proprotein convertase subtilisin/kexin type 9; PFIC, progressive familial intrahepatic cholestasis; PPI, proton pump inhibitor; RKI, Robert Koch Institute; SmPC, summary of product characteristics; SSNRI, selective serotonin–norepinephrine reuptake inhibitor; SSRI, selective serotonin reuptake inhibitor; TACE, transarterial chemoembolization.

## 4 Discussion

In this study, we analyzed a total of 438 queries received by the clinical‐pharmacological DIC of Hannover Medical School over a period of 33 months. Even though the sample size was around 26% smaller than in our previous investigation (study period from October 2018 to April 2022) [2], the number of queries handled per month by our DIC remained relatively constant over time (13.3 vs. 13.8 queries per month, respectively). The sample size of the present study lay within the range of other analyses in the field, which displayed sample sizes between 55 and 744 queries [3, 9, 10, 11, 13, 14, 15, 16, 17, 26, 28, 29, 30, 31, 32]—and was considered appropriate for an update from our DIC.

Interestingly, we found several similarities but also some notable differences between our present and previous research [2]. The proportion of patient‐specific queries was comparable (85.2% vs. 82.8% [2]), physicians were the largest group of inquiring healthcare professionals in both studies (95.7% vs. 96.1% [2]), and also the top three medical specialties that most frequently consulted our DIC (i.e., internal medicine, psychiatry and psychosomatic medicine, and surgery) remained the same. Physicians also represented the group of healthcare professionals who predominantly consulted DICs in other analyses [8, 9, 14, 23, 26, 31], and internists in particular were among the main users of DICs located in Dresden [28] and Munich, Germany [32]. Consistent with our earlier research [2] but diverging from an older investigation by Tröger and Meyer (Magdeburg, Germany), we received a notable proportion of queries (16.0%) from surgeons. This substantial percentage may point to the growing interdisciplinary collaboration between medical and surgical disciplines that has developed at Hannover Medical School in recent years [33].

The elevated level of specialization at our tertiary care university hospital likely explains the consistently high proportion of patient‐specific queries (exceeding 80%). This observation is in line with an analysis by Nilsson et al. from a university hospital‐affiliated Swedish DIC jointly operated by clinical pharmacologists and pharmacists (79% patient‐specific queries) [31], but it contrasts with studies by Almazrou et al. [34] and Tefera et al. [11] conducted at pharmacist‐run Saudi Arabian and Ethiopian DICs, in which the proportions of patient‐specific queries were notably lower (44% and 53.1%, respectively). While responses to general queries typically aim to provide factual DI, responses to patient‐specific queries focus on individually tailored treatment strategies. These responses offer critical assessments of the advantages and disadvantages of specific pharmacological therapies in individual patient scenarios, considering patient‐specific factors such as age, sex, disease severity, comorbidities, and comedication. Delivering factual DI (e.g., details on drug availability, stability, compatibility, interactions, and ADRs) is a core competency of pharmacists. Conversely, evaluating the benefit–risk profile of pharmacological therapies within the context of individual patients is the domain of physicians. These professional distinctions may account for the disparities observed between the studies by Almazrou et al. [34] and Tefera et al. [11] (both pharmacist‐led) and our investigation (physician‐led). Intriguingly, a recent analysis of a university hospital pharmacy‐operated DI service in Munich, Germany, revealed proportions of patient‐specific queries and queries from physicians (91% and 95%, respectively) that were comparable to our study, but focused exclusively on ADRs [32]; other DI query categories were not assessed.

The high degree of specialization at our clinic is underscored by the queries presented and discussed in Table 3, three of which dealt with the pharmacological management of rare diseases (progressive familial intrahepatic cholestasis type 1, factor VII deficiency, and chronic Q fever). It should be noted, however, that the cases showcased in Table 3 were selected for illustrational and educational purposes only; they were not intended to be representative of the entire sample of queries analyzed in the present investigation. Timpe and Motl showed that the complexity of DI queries increased between 1995 and 2004 [35], and 70% of respondents in a survey conducted among US DICs reported an increase in the number of complex DI queries between 2003 and 2008 [19]. To this point, only a minority of researchers have showcased specific DI queries in their publications [7, 16, 17, 22, 36]. These case presentations were either in condensed form [7, 22] or lacked the corresponding DIC responses [16, 17]. With the exception of Lumpe et al.'s 1998 article [36] and our 2022 publication [2], our study stands out as the sole report that presents and provides commentary on a curated selection of clinically intricate DI queries, which can be regarded as a strength of our work.

Remarkably, the proportion of queries from academic teaching hospitals declined by approximately one‐half between our previous research and the current investigation (13.0% [2] vs. 6.4%). In line with a recent systematic review [37], this may point to an increasing workload in peripheral clinics (e.g., due to a shortage of physicians and nurses), leaving little time to consult external specialists during the treatment process.

Whereas the mean age of patients in our study was comparable with our previous research (54.0 ± 22.4 years and 55.6 ± 22.9 years [2]), the proportion of female patients increased (60.3% vs. 51.4% [2]). Of note, while the age distribution in our previous study showed a bimodal curve with peaks at 21–30 years and 61–70 years [2], there was a single peak in the present investigation at 51–70 years. The three most frequently addressed query categories (i.e., indications/contraindications, ADRs, and PDIs) corresponded well to the DI needs most commonly cited by healthcare professionals in a recent systematic literature review [37], and they remained stable between our previous investigation [2] and the current analysis. On the other hand, we detected certain changes in the differences between patient‐specific and general queries. As compared to general queries, patient‐specific queries were more often related to ADRs, PDIs, indications/contraindications, posology/dose adjustment, and TDM (before adjusting for multiple comparisons), whereas in our previous study, we detected differences between general queries and patient‐specific queries only with respect to ADRs, PDIs, and PKIs (also without adjustments for multiple testing). Of note, in the present investigation, only the differences between general and patient‐specific queries with regard to indications/contraindications and PDIs remained statistically significant after application of the Holm–Bonferroni correction method to address the problem of multiple comparisons and to reduce the likelihood of type I errors. These findings may be explained by the fact that general information on ADRs, indications/contraindications, posology/dose adjustment, TDM, and drug–drug interactions can be relatively easily accessed by healthcare professionals independently. A systematic literature review by Tan et al. highlighted the variety of DI sources used by healthcare professionals, the most popular of which were reference books, journals, drug databases, textbooks, and product‐specific information (e.g., SmPCs), only to name a few [37]. However, for the transfer of general clinical‐pharmacological knowledge to the treatment of individual patients, consultation and discussion with human experts (e.g., via a DIC) is still paramount. A Swedish questionnaire study revealed that 85% of responding physicians searched for DI at least weekly [38]. At the same time, healthcare practitioners often lack the time and also the expertise to address complex DI queries independently [23]. While access to scientific literature and other references has improved significantly in recent decades, mainly due to increased online search capabilities, the volume of available information on any given pharmacological topic has grown substantially [23]. Moreover, the complexity of DI queries has markedly increased in recent years [23]. Consequently, the time required to handle DI queries has not diminished significantly compared to earlier periods [23], indicating a continued need for DICs to support clinicians in their daily practice. In the present study, we did not systematically assess the time required to answer DI queries, which represents a limitation of our investigation. From our experience, typically 30–120 min are needed to answer a DI query.

Pharmacotherapy in older adults is particularly challenging due to altered pharmacokinetics and pharmacodynamics compared to younger individuals [39]. At our DIC, the proportion of queries related to pharmacotherapy in advanced age increased from 6.1% [2] to 9.1%, which may reflect Germany's demographic shift towards an aging population: currently, over one‐fifth of Germans are 65 years or older [40], with this percentage expected to increase in the coming decades [41]. Other studies in the field did not examine pharmacotherapy in advanced age as a distinct topic [10, 11, 14, 29, 34, 35, 42] but similar demographic trends are anticipated in emerging nations, too [43]. Consequently, DICs in emerging nations may soon experience a comparable rise in queries related to geriatric pharmacotherapy.

The utility of clinical‐pharmacological advice on drug use during pregnancy was highlighted by Erdeljić et al., who showed that clinical pharmacologists' assessments were more accurate than the US Food and Drug Administration classification system in predicting pregnancy outcomes after pregnancy‐related drug exposures [44]. Furthermore, in a survey of physicians who sought guidance from a Norwegian DIC on drug use during pregnancy, 9% of respondents reported that the information provided by the DIC had averted termination of an intended pregnancy [25]. Remarkably, the proportion of queries related to pregnancy or breastfeeding at our DIC rose from 3.5% [2] to 6.2%. Even though this percentage is still considerably lower than in older reports by Llerena et al. (drug use in pregnancy: 18%; drug use in breastfeeding: 4%) [3] and Schwarz et al. (drug use in pregnancy/lactation: 16%) [28], it is notably higher than in a more recent study by Tefera and colleagues (drug use in pregnancy: 2.6%) [11]. This indicates that while a general decline in DI queries related to drug use during pregnancy or breastfeeding has been observed in the last decades, presumably due to the easy and free‐of‐charge access to online databases such as Embryotox [45], Bumps (Best Use of Medicines in Pregnancy) [46], and the Drugs and Lactation Database [47], consultation with experts in clinical pharmacology is still considered useful by attending healthcare professionals, especially in clinically complex cases (e.g., case no. 3 in Table 3).

The number of queries related to pharmacogenetics and TDM in our study appeared relatively small. At Hannover Medical School, similar to the majority of university hospitals in Germany, pharmacogenetic tests and TDM are technically conducted by the Central Laboratory (which in the case of Hannover Medical School is affiliated with the Institute for Clinical Chemistry, not with the Institute for Clinical Pharmacology, at which the DIC is located). The Central Laboratory also provides explanations and interpretations of pharmacogenetic and TDM test results, which may be sufficient for healthcare professionals in most routine cases. We hypothesize that only in special, therapeutically challenging pharmacogenetic or TDM cases, the DIC is contacted by clinicians as an additional source of information and advice, potentially explaining the comparatively low number of pharmacogenetic and TDM queries in our study.

Besides its single‐center and retrospective design, the primary limitation of our study is the absence of data on patient‐related outcomes. Consequently, it was not possible to ascertain whether the consultation of the DIC by attending healthcare professionals resulted in benefits for their patients. To examine this aspect, a control group would have been necessary, which was not feasible within the constraints of this study—a drawback our investigation shares with many other DIC studies [8, 9, 10, 11, 13, 14, 15, 16, 17, 18, 22, 26, 27, 28, 31, 32, 34, 42]. Moreover, the absence of data on how often the recommendations of the DIC were adhered to and implemented into clinical practice (i.e., acceptance rates), or how satisfied the inquiring healthcare professionals were with the DIC recommendations represent substantial limitations of our work. Due to the hypothesis‐generating, non‐confirmatory nature of our study, the results of the inferential statistical tests—despite adjustments for multiple comparisons—must be interpreted with considerable caution.

In summary, our study suggests that query characteristics at our DIC remained relatively stable between the time periods 2018–2022 [2] and 2022–2024, especially with respect to type of queries (> 80% patient‐specific), origin of queries (mainly from within Hannover Medical School itself), profession of inquiring healthcare professionals (> 90% physicians), medical specialties of inquirers (top three: internal medicine, psychiatry and psychosomatic medicine, surgery), and query categories (top three: indications/contraindications, ADRs, PDIs). Nonetheless, we also observed certain changes between our previous research [2] and the current investigation, most notably higher proportions of queries related to pharmacotherapy in advanced age and drug use during pregnancy or breastfeeding, as well as a unimodal instead of a bimodal age distribution curve among patient‐specific queries. Besides time‐dependent changes, fundamental differences in query characteristics across DICs may also stem from country‐specific factors, the affiliation and service area of DICs, and the composition of DIC staff (clinical pharmacologist‐led vs. pharmacist‐led) [2]. We propose that DICs can effectively contribute to high‐quality patient care and enhanced pharmacotherapy safety [2]. Future research should focus on prospective controlled studies to determine if DICs genuinely improve clinically relevant patient outcomes [2].

## Author Contributions

**Johannes Heck:** conceptualization; data curation; investigation; methodology; formal analysis; visualization; writing – original draft; writing – review and editing. **Dirk O. Stichtenoth:** writing – review and editing; supervision. **Christoph Schröder:** writing – review and editing. **Ruxandra Sabau:** writing – review and editing. **Anna‐Leena K. Heim:** formal analysis; writing – review and editing. **Felix Koop:** formal analysis; writing – review and editing. **Thorben Pape:** formal analysis; writing – review and editing. **Sebastian Schröder:** formal analysis; writing – review and editing. **Martin Schulze Westhoff:** formal analysis; writing – review and editing. **Benjamin Krichevsky:** methodology; formal analysis; writing – review and editing. **Martin Klietz:** writing – review and editing. **Stephan Greten:** writing – review and editing. **Carsten Schumacher:** methodology; formal analysis; writing – original draft; writing – review and editing.

## Funding

The authors have nothing to report.

## Conflicts of Interest

The authors declare no conflicts of interest.

## Supporting information


**Table S1:** Query categories, stratified by specialization of inquiring healthcare professionals (assignment of more than one category per query was possible).

## Data Availability

The data that support the findings of this study are available upon reasonable request from the corresponding author.
