# Supplementary material for: Clinical‐Pharmacological Drug Information Center of Hannover Medical School: Update From a Tertiary Care University Hospital (2022–2024)
Source: Pharmacol Res Perspect. 2026 Apr 8;14(2):e70247. doi: 10.1002/prp2.70247 (PMC13061747; doi:10.1002/prp2.70247)
Supplement: Supplementary file 1 — Table S1: Query categories, stratified by specialization of inquiring healthcare professionals (assignment of more than one category per query was possible). [file PRP2-14-e70247-s001.docx]

**SUPPLEMENTARY TABLE 1** Query categories, stratified by specialization of inquiring healthcare professionals (assignment of more than one category per query was possible)

| **Specialization of inquiring healthcare professional** | **Total no. of submitted queries** | **Query categories** | | | | | | | | | |
| --- | --- | --- | --- | --- | --- | --- | --- | --- | --- | --- | --- |
|  |  | **Adverse drug reaction—no. (%)** | **Indication/**  **contraindi­cation—no. (%)** | **Posology/dose adjustment (e.g., due to renal or hepatic insuffi­ciency)—no. (%)** | **Therapeutic drug monitoring—no. (%)** | **Pharmaco­genetics—no. (%)** | **Pharmaco­dynamic interaction—no. (%)** | **Pharmaco­kinetic interaction—no. (%)** | **Pregnancy and breast­feeding—no. (%)** | **Pharmaco­therapy in advanced age—no. (%)** | **Other—no. (%)** |
| Internal medicine | 125 | 53 (42.4) | 56 (44.8) | 30 (24.0) | 19 (15.2) | 3 (2.4) | 44 (35.2) | 58 (46.4) | 4 (3.2) | 8 (6.4) | 18 (14.4) |
| Psychiatry and psychosomatic medicine | 77 | 45 (58.4) | 41 (53.2) | 14 (18.2) | 8 (10.4) | 9 (11.7) | 26 (33.8) | 20 (26.0) | 2 (2.6) | – | 8 (10.4) |
| Surgery | 70 | 21 (30.0) | 24 (34.3) | 14 (20.0) | 4 (5.7) | – | 38 (54.3) | 27 (38.6) | – | 22 (31.4) | 2 (2.9) |
| Urology | 41 | 14 (34.1) | 21 (51.2) | 24 (58.5) | 10 (24.4) | 1 (2.4) | 18 (43.9) | 17 (41.5) | – | 7 (17.1) | 4 (9.8) |
| Gynecology and obstetrics | 40 | 11 (27.5) | 21 (52.5) | 5 (12.5) | 3 (7.5) | 4 (10.0) | 25 (62.5) | 23 (57.5) | 13 (32.5) | – | 3 (7.5) |
| Neurology | 17 | 9 (52.9) | 8 (47.1) | 3 (17.6) | 4 (23.5) | 2 (11.8) | 9 (52.9) | 8 (47.1) | – | 1 (5.9) | 2 (11.8) |
| Pediatrics | 13 | 5 (38.5) | 7 (53.8) | 3 (23.1) | – | 2 (15.4) | 7 (53.8) | 7 (53.8) | – | – | 2 (15.4) |
| Radiology and radiotherapy | 12 | 12 (100) | 8 (66.7) | – | – | – | 2 (16.7) | 1 (8.3) | – | – | 5 (41.7) |
| Other | 37 | 10 (27.0) | 13 (35.1) | 8 (21.6) | 4 (10.8) | – | 12 (32.4) | 14 (37.8) | 7 (18.9) | 2 (5.4) | 9 (24.3) |
| Not documented | 6 | 6 (100) | 3 (50.0) | – | – | – | 1 (16.7) | 1 (16.7) | 1 (16.7) | – | – |

Abbreviations: no., number
